# Enhancement of Calcium Ion Permeation via Resonant Coupling of Ion and Terahertz Waves in Voltage Gated Calcium Channels

**DOI:** 10.1002/advs.202520475

**Published:** 2026-02-19

**Authors:** Zihao Zhang, Yuankun Sun, Xinqiao Zhou, Sunchao Huang, Shengpeng Yang, Shaomeng Wang, Yubin Gong

**Affiliations:** ^1^ School of Electronic Science and Engineering University of Electronic Science and Technology of China Chengdu China; ^2^ National Key Laboratory of Science and Technology on Vacuum Electronics University of Electronic Science and Technology of China Chengdu China; ^3^ Terahertz Radiation and Application Key Laboratory of Sichuan Province University of Electronic Science and Technology of China Chengdu China

**Keywords:** ion flux, resonant coupling, terahertz, voltage gated calcium channels

## Abstract

Dysregulated calcium ion (Ca^2+^) influx is implicated in diverse channelopathies. Terahertz (THz) waves have been explored as a promising approach to modulate the influx, which primarily target resonant interactions with chemical groups in channels. Here, we demonstrate a strategy that directly regulates the motion of confined Ca^2+^ ions within the selectivity filter via resonant THz waves. We characterize the ions' axial oscillations, identifying a distinct intrinsic frequency of 1.65 THz and two coherent modes. By tuning a THz electric field to this frequency, we induce a remarkable resonance excitation that lowers the energy barrier between binding sites, achieving a statistically significant enhancement of Ca^2+^ permeation in our model. Meanwhile, we show that the degree of coherence is precisely tunable by the resonant THz field and temperature. Furthermore, quantum mechanics analyses reveal transition frequencies and wavefunctions that validate the observed oscillation modes, confirming that the collective motion exhibits discrete quantum eigenstates. Our results introduce a proof‐of‐concept, ion‐targeted strategy for manipulating Ca^2+^ permeation and propose a theoretical coherence mechanism for high‐flux ions transport. This work advances the understanding of ion channel physics via a mechanism‐oriented framework that lays the groundwork for exploring novel bio‐electromagnetic modulation.

## Graphical abstract

We introduce a novel, ion‐targeted strategy to enhance calcium permeation in voltage gated calcium channels. By directly driving the ions’ coherent collective motion via resonant coupling with a 1.65 THz field, we lower the inter‐site energy barrier, inducing a statistically significant enhancement of ion influx. Furthermore, this work offers a theoretical perspective of quantized collective motion mechanism for ions high‐flux transport.

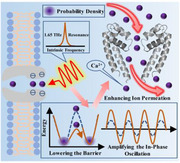

## 1 Introduction

Voltage‐gated calcium channels (Ca_v_) are essential transmembrane proteins that mediate the influx of calcium ions (Ca^2+^) into cells in response to membrane depolarization [1, 2]. Ca^2+^ ions serve as critical second messengers, triggering a wide array of fundamental physiological processes, including muscle contraction, gene expression, neurotransmitter release, and diverse cellular responses [3, 4]. Given their central role in cellular excitability and signaling, dysfunctions in Ca_v_ channels are directly linked to a wide spectrum of disorders, known as channelopathies, such as cardiac arrhythmias, epilepsy, chronic pain, and ataxia [5, 6, 7, 8]. Deciphering the precise interplay between channel structure and ion transport is instrumental not only for unraveling the biophysical mechanisms underlying these channelopathies but also for establishing precision strategies to modulate channel function. Extensive studies have identified the selectivity filter (SF) as the core functional domain governing ion permeation in Ca_v_ channels [9, 10, 11, 12, 13]. The SF is characterized by a highly conserved ring of four glutamate residues (the EEEE locus), whose negatively charged carboxylate side chains form a high‐affinity Ca^2+^ binding site. This structure underpins the channel's ability to selectively permeate Ca^2+^ over the more abundant sodium ions [14, 15, 16]. Therefore, manipulating the SF is an effective method to regulate calcium fluxes, whether correcting abnormal permeation or enhance the flow to induce rapid apoptosis of abnormal cells [17].

Terahertz (THz, 10^12^ Hz) radiation, situated between the microwave and infrared regions of the electromagnetic spectrum, is emerging as a promising tool for manipulating physiological processes and advancing non‐invasive bio‐interventions. Its potential stems from the fact that the THz frequency range corresponds to the characteristic frequencies of crucial intra‐ and inter‐molecular motions, such as the vibrations of hydrogen bonds and van der Waals forces [18, 19, 20, 21, 22, 23, 24, 25, 26]. Consequently, THz waves have been investigated for modulating a variety of biological activities, including neuronal function, learning, and brain activity, with a particular focus on the manipulation of ion channels [27, 28, 29, 30, 31, 32, 33]. Both experimental and computational studies have demonstrated the feasibility of modulating ion channel function with THz waves. Experimentally, a 53.53 THz wave was shown to increase potassium current and alter action potential waveforms [27]. Numerous simulation studies have further elucidated the underlying mechanisms. For instance, in potassium channels, a 51.87 THz field was found to enhance K^+^ permeation in the KcsA channel by resonating with carbonyl groups in the SF [34, 35], whereas a 36.75 THz field was reported to decrease the structural stability of the K_V_1.2 channel [36]. In the case of calcium channels, a 42.55 THz wave was shown to improve both the selectivity and conductance of Ca_v_Ab (Ab, Arcobacter butzleri) by resonating with the stretching mode of either the ‐COO^−^ or the ‐C = O group [37]. Simulation and experimental results revealed that 42.5 THz waves enhanced Ca^2+^ conductance in Ca_v_1.2 by resonating with the stretching mode of the ‐COO^−^ group in the selectivity filter [38]. For sodium channels, a 48.20 THz field was found to increase ion permeation by a mechanism in which carboxylate groups absorb energy and transfer it to the permeating sodium ions [39].

However, the aforementioned studies primarily focused on how THz radiation influences chemical groups within the channel protein, while largely overlooking the dynamics of the permeating ions themselves. In fact, advances in high‐resolution structural biology and computational modeling suggest that the collective movement and dynamic interplay of ions are fundamental to both high‐flux permeation and selectivity [40, 41, 42, 43, 44, 45]. Foundational models, such as the two‐ion pore model in Ca_v_ channels, posit that the simultaneous occupancy of the selectivity filter by two ions is a prerequisite for efficient permeation and monovalent ion blockade [46, 47]. This concept is further refined by the three‐ion knock‐on mechanism, identified through all‐atom molecular dynamics (MD) simulations, where an incoming third Ca^2+^ ion displaces the original ions to facilitate influx [48]. This knock‐on mechanism, driven by strong inter‐ionic electrostatic interactions, is considered a cornerstone of high‐flux transport and has also been observed in sodium and potassium channels [49, 50, 51, 52]. More recent evidence suggests that ionic behavior in the confined SF environment transcends simple stochastic motion. Studies on KcsA potassium channels, for example, have revealed that collectively permeating ions exhibit significant coherent oscillations at their binding sites, which may serve as a directional guide for efficient transport [53]. Critically, studies on Na^+^ and K^+^ channels have demonstrated that THz waves can enhance permeation by directly manipulating the collective motion of the ions through resonance, achieved by tuning the wave frequency to match the ions' intrinsic oscillations [54]. To date, however, a similar investigation into the intrinsic dynamics of Ca^2+^ ions and their potential modulation by resonant THz fields remains unexplored. Therefore, characterizing the dynamics of Ca^2+^ within the SF and identifying a resonant frequency to modulate its permeation holds great significance for both fundamental Ca_v_ research and future biomedical applications.

In this study, we systematically investigated the dynamics of Ca^2+^ ions within the SF of the equivalent Ca_v_Ab channel and explore their modulation by resonant THz fields. Although Ca_v_Ab is a bacterial homolog, it serves as a well‐established structural surrogate for mammalian voltage‐gated calcium channels (specifically the Ca_v_1 and Ca_v_2 families) due to their shared, highly conserved EEEE locus within the selectivity filter. This structural homology implies that the physical mechanisms of ion dynamics governed by this motif are relevant to understanding human Ca_v_ physiology and associated channelopathies. Using Langevin dynamics, we simulated the motion of ion pair confined within the two potential wells formed by the EEEE locus, successfully identifying the intrinsic oscillation frequencies of the system. Building on these findings, we demonstrated that an external THz field tuned to the resonant frequency of 1.65 THz significantly enhanced ion oscillation and reduced the permeation energy barrier. We further characterized the collective behavior of the ion pair, revealing distinct in‐phase (1.65 THz) and out‐of‐phase (2.84 THz) coherent oscillation modes, and systematically evaluated how their coherence is influenced by THz fields and temperature. To provide a deeper physical understanding, we modeled the confined ion pair as a two‐dimensional coupled quantum harmonic oscillator (CQHO). The quantum mechanical analysis yielded transition frequencies and wavefunction probability distributions that are in excellent agreement with our classical dynamics simulations, thereby validating the observed frequencies and oscillation modes. Collectively, this work establishes a novel theoretical methodology for manipulating Ca_v_ channels by directly targeting the motion of confined Ca^2+^ ions and provides a scientific framework that bridges the classical and quantum mechanical descriptions of ion dynamics in channels.

## 2 Results

### 2.1 Dynamics of Confined Ca^2+^ Ions

#### 2.1.1 Oscillation Modes of Confined Ca^2+^ Ions at Binding Sites

**FIGURE 1:**
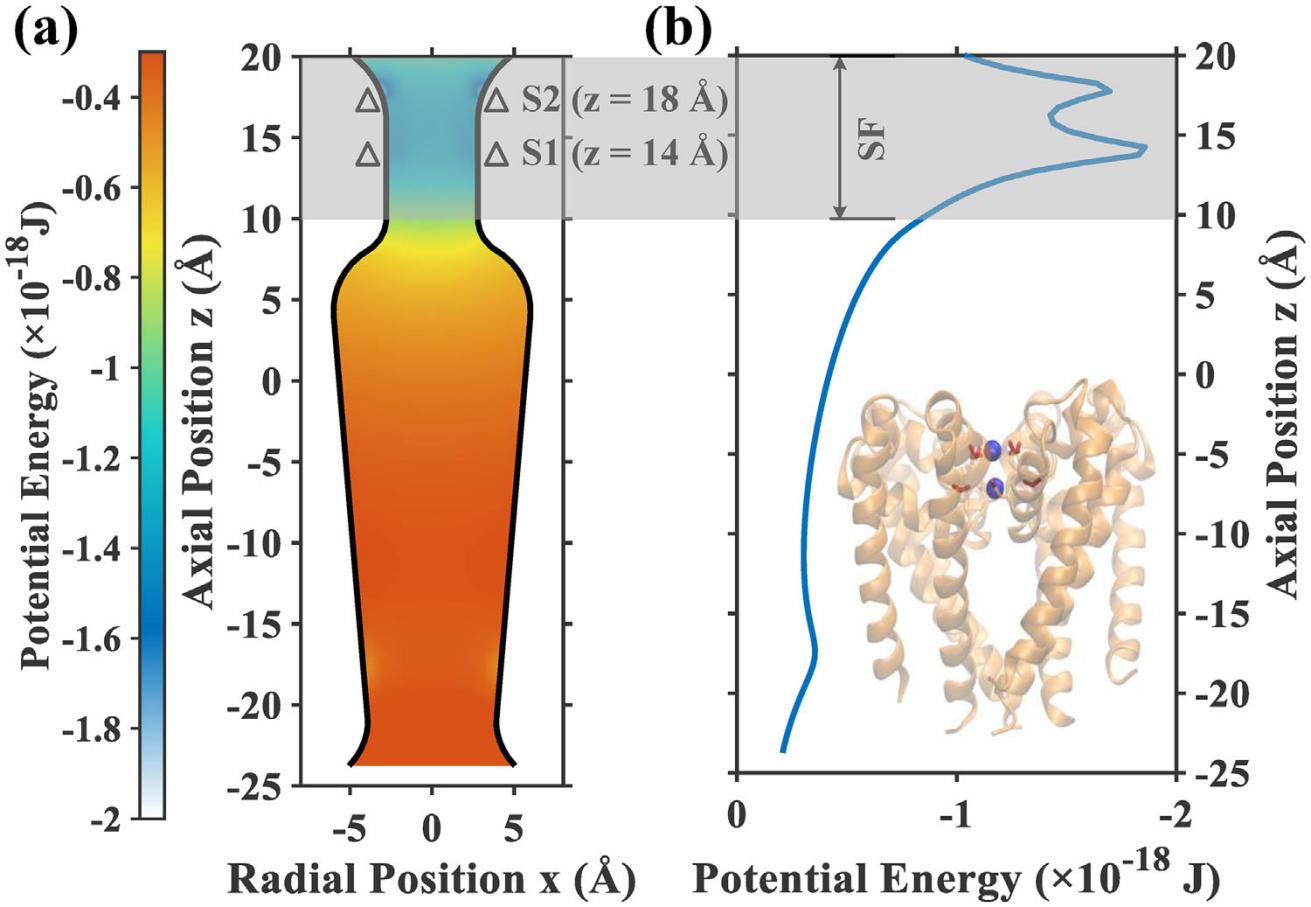
Potential energy landscape and structural model of the Ca_v_Ab channel. (a) The 2D potential distribution on the longitudinal cross‐section (XOZ plane). The grey shaded region highlights the selectivity filter (SF). Triangles (Δ) indicate the locations of the EEEE locus, corresponding to binding site 1 (S1, z = 14 Å) and binding site 2 (S2, z = 18 Å). (b) The 1D potential energy profile along the channel axis aligned with the 2D map. The inset displays the structural model (PDB ID: 4MVQ), where blue spheres represent Ca^2+^ ions and red sticks represent the negatively charged carboxyl groups, illustrating the structural basis of the potential wells. The z‐axis is oriented from the extracellular (positive z) to the intracellular (negative z) side.

Our simulations focused on the Ca_v_Ab channel, which features binding sites formed by highly symmetrical residues. The primary affinity for Ca^2+^ ions within the selectivity filter (SF) is known to be dominated by electrostatic attraction from the negatively charged carboxyl (‐COO^−^) groups of glutamate residues, with comparatively minor contributions from uncharged carbonyl (‐C = O) groups [37]. Consequently, our model centers on the two principal, high‐affinity binding sites provided by two distinct EEEE locus. By incorporating the Coulomb forces from these residues, as well as the polarization charges arising from dielectric difference between proteins on the SF wall and environment inside the channel, we calculated the static electrostatic potential landscape of the channel, as depicted in Figure 1. The structural stability of the selectivity filter which justifies the approximation of the binding site environment as an average potential energy landscape, was verified by a 100‐ns all‐atom molecular dynamics simulation (detailed in Note S4, Supporting Information). The 2D potential map (Figure 1a) illustrates the channel's geometry. The selectivity filter (SF) is highlighted by the grey shaded region spanning from z = 10 Å to z = 20 Å. Within this restricted zone, triangles (Δ) explicitly mark the axial locations of the glutamate residues (EEEE locus) that form the two high‐affinity binding sites at z = 14 Å and z = 18 Å. The corresponding 1D potential energy profile along the central axis is presented in Figure 1b, aligned vertically with the 2D map. This profile clearly reveals two deep potential wells corresponding to the marked binding sites. To visualize the physical origin of these wells, a structural model of the Ca_v_Ab channel (PDB ID: 4MVQ) is included as an inset in Figure 1b, showing the Ca^2+^ ions (blue spheres) confined by the carboxyl groups (red sticks) within the SF [13]. Additionally, a much shallower potential well is present near z = −17 Å. This feature, generated by four dipoles as described in previous work to guarantee the permeation [9, 55], was retained for model integrity, though its impact on the dynamics within the high‐affinity SF region is considered negligible.

**FIGURE 2:**
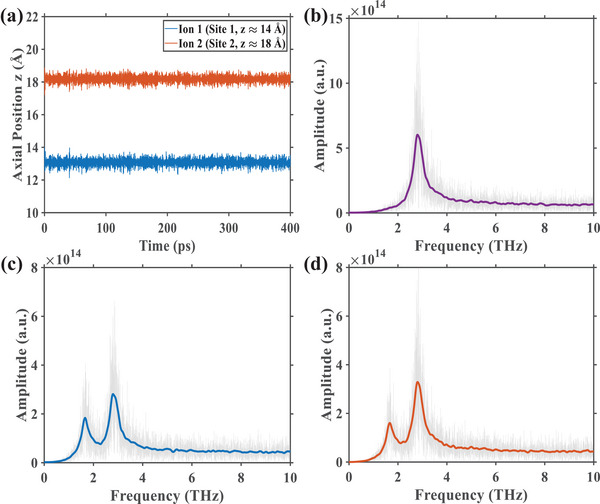
Time and frequency domain analysis of the two‐ion system axial dynamics. (a) Axial trajectories of the two Ca^2+^ ions, showing ion 1 (blue) and ion 2 (vermilion) localized at binding site 1 and binding site 2, respectively, over the 400 ps simulation period. (b) Spectrum of the inter‐ionic distance (|z_1_‐z_2_|), highlighting the out‐of‐phase vibrational mode of the ion pair. (c, d) Spectra of the axial acceleration for ion 1 and ion 2, respectively. The peaks reveal the dominant vibrational frequencies (1.65 and 2.84 THz) for each individual ion. In panels (b‐d), the light traces represent the raw spectral data, while the solid lines are the corresponding Gaussian‐smoothed curves.

Based on the potential energy landscape, we employed the Langevin dynamics to simulate the motion of the two confined Ca^2+^ ions. Our analysis focuses on the axial (z‐axis) dynamics, as this is the direction of ion permeation, while ignoring the radial (x, y‐axis) motions. The Langevin equation holistically describes the forces governing ionic motion, comprising: a conservative force (*F_cons_*), which includes electrostatic and short‐range interactions with the channel and the other ion; a frictional force characterized by a damping rate (γ), representing drag from water molecules; and a stochastic force (ξ(*t*)), arising from random thermal collisions [9]. The explicit derivations are detailed in Notes S1–S3. Solving this equation at an ambient temperature of 310 K with a 2.5 fs time step yielded the axial trajectories shown in Figure 2a. The time‐domain results confirm that ion 1 (blue) and ion 2 (vermilion) are tightly confined to their respective binding sites (site 1 at z = 14 Å and site 2 at z = 18 Å), oscillating around mean positions of z ≈ 13.1 Å and z ≈ 18.2 Å. This slight displacement from the static potential minima is attributed to the Coulombic repulsion between the two cations. To identify the characteristic oscillation frequencies, we applied a Fast Fourier Transform (FFT) to the axial acceleration of the ions. The resulting spectra for ion 1 (Figure 2c) and ion 2 (Figure 2d) are nearly identical, each prominently displaying two distinct peaks at 1.65 THz and 2.84 THz. These peaks signify two fundamental modes of axial oscillation for the ion pair. The spectrum of the inter‐ionic distance, shown in Figure 2b, exhibits a single dominant peak at 2.84 THz. This strongly suggests that the 2.84 THz frequency corresponds to the out‐of‐phase motion of the ion pair, while the 1.65 THz frequency represents the in‐phase motion of the ions oscillating within their respective potential wells.

**FIGURE 3:**
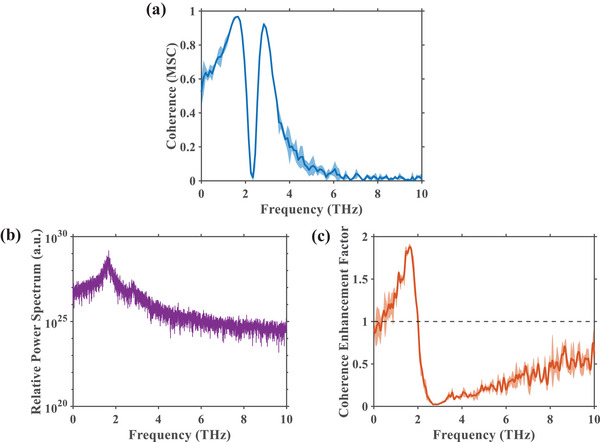
Statistical coherence analysis of the two‐ion axial motion. (a) Magnitude Squared Coherence (MSC) between the axial velocities of the two ions, indicating strong kinematic correlation in specific frequency bands. (b) Spectrum of the coherent sum of the ions' axial accelerations (S_coh_), plotted on a logarithmic scale. (c) Radiative coherence enhancement factor, calculated as the ratio of the coherent power spectrum (S_coh_) to the sum of the individual incoherent power spectra (S_incoh_). Values greater than the baseline of 1 (dashed line) indicate constructive interference (in‐phase motion), while values less than 1 indicate destructive interference (out‐of‐phase motion). Data are presented as the mean ± standard (shown as shaded bands) deviation derived from three independent simulations, confirming the stability of these collective modes.

Beyond the dynamics of individual ions, we next investigated the nature of their coupled motion. To quantify the phase stability between the ions, we first calculated the Magnitude Squared Coherence (MSC) of their axial velocities, which describes the degree of linear correlation between two velocities at different frequencies. To ensure robustness, the MSC spectrum was averaged over three independent simulation trajectories. The resulting MSC spectrum, presented in Figure 3a, exhibits two peaks with mean values approaching unity: ∼0.96 at 1.65 THz and ∼0.92 at 2.84 THz. These high MSC values confirm a highly stable phase relationship between the two ions at these specific oscillation frequencies. To determine the precise nature of this phase relationship, we employed an analysis based on the concept of electromagnetic radiation generated by the accelerating ions. We defined the coherent radiation power based on the square of the sum of the ions' accelerations and the incoherent power based on the sum of their individual squared accelerations. The ratio of these two quantities yields the radiative coherence enhancement factor. This factor provides a clear distinction between oscillation modes: a value approaching 2 indicates perfectly in‐phase motion, a value near 0 signifies perfectly out‐of‐phase motion, and a value of 1 implies uncorrelated motion. As shown in Figure 3c, the mean enhancement factor reaches approximately 1.91 at 1.65 THz and drops to about 0.12 at 2.84 THz. This result, corroborated by the strong peak in the coherent radiation spectrum at 1.65 THz (Figure 3b), identifies the 1.65 THz mode as a coherent in‐phase oscillation and the 2.84 THz mode as a coherent out‐of‐phase oscillation of the ion pair.

#### 2.1.2 Transmembrane Current of the Proposed Model

To demonstrate a macroscopic view of this equivalent Ca_v_Ab channel, we conducted a permeation simulation using a multi‐ion channel model flanked by two reservoirs. A reset boundary condition was employed at the channel exit (z = −25 Å); upon crossing this trigger, an ion was repositioned in the right‐side reservoir to maintain a constant number of ions in the system. Simulations were initiated with two ions occupying the binding sites within the selectivity filter and a third ion placed randomly in the right reservoir. Under a constant −400 mV transmembrane potential, ions from the reservoir are driven toward the filter, inducing a “knock‐on” mechanism where Coulombic repulsion dislodges the bound ions, facilitating permeation.

While this voltage magnitude is elevated compared to typical physiological conditions, it was employed here as a proof‐of‐concept strategy to accelerate ion permeation events within the accessible simulation timescales and to maximize the signal‐to‐noise ratio for further identifying resonance effects. In our baseline simulations (five independent 60 ns trajectories), we observed a mean of 0.40 ± 0.55 permeation events, corresponding to an average ionic current of approximately 2.13 pA, within the range of maximum current 7.5 pA from the previous Brownian dynamics for Ca_v_Ab channels [9]. The calculated channel conductance is ∼5.33 pS, which remains on the same order of magnitude as the experimental channel conductance values of 8–9 pS [56, 57].

### 2.2 Regulation of Ions’ Motion via THz Waves

#### 2.2.1 Enhancement of Ions Permeation by Resonant THz Waves

**FIGURE 4:**
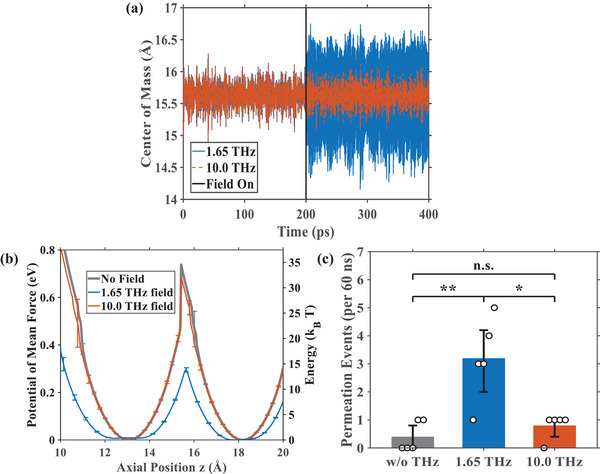
Effect of external THz‐EMW on the dynamics, potential energy landscape, and permeation statistics of the two‐ion system. (a) Axial trajectory of the two‐ion center of mass under resonant (1.65 THz, blue) and non‐resonant (10.0 THz, vermilion) field conditions (0.3 V/nm). The external field is applied at t = 200 ps. The resonant field significantly excites the axial oscillation, increasing its amplitude compared to the non‐resonant case. (b) 1D Potential of Mean Force (PMF) profiles along the z‐axis under no‐field (gray), resonant (1.65 THz, blue), and non‐resonant (10.0 THz, vermilion) conditions (0.3 V/nm). Data are averaged from three independent simulations, with error bars representing the standard deviation. The right y‐axis displays energy in units of *k_B_T* at 310 K. The resonant field significantly lowers the energy barrier between the two binding sites (potential wells at z ≈ 13.1 Å and z ≈ 18.2 Å). (c) Statistical analysis of Ca^2+^ permeation events under 3 V/nm electric field. The bar chart displays the average number of permeation events observed over a 60‐ns duration under three conditions: without THz field (gray), with a resonant 1.65 THz field (blue), and with a non‐resonant 10.0 THz field (vermilion). Data are presented from five independent simulation trajectories per condition. Open circles represent individual counts, and error bars indicate the bootstrap 95% confidence interval (CI) of the mean (10 000 resamples). Statistical significance between groups was assessed using an Exact Poisson test (two‐sided conditional binomial test on total event counts). Double asterisks (**) indicate a statistically significant difference with *p* < 0.01. The 1.65 THz field induces a statistically significant enhancement in ion flux compared to the control. The effect size is quantified by the Rate Ratio (RR), showing a statistically significant enhancement in ion flux compared to the control (RR = 8.0, 95% CI: 1.88–71.72, for Resonant vs. Control, see Statistical Analysis in Section Methods for details).

Having identified the intrinsic oscillation modes of the ion pair, we next sought to determine if these dynamics could be externally modulated to influence ion permeation. To this end, we first applied a z‐directed THz electric field (0.3 V/nm) to the system, with its frequency tuned to the in‐phase mode at 1.65 THz. The effects of this resonant field, contrasted with a non‐resonant 10.0 THz field, are presented from both dynamic and energetic perspectives in Figure 4. From a dynamic standpoint, Figure 4a displays the axial trajectory of the two‐ion center of mass. Upon application of the external field at t = 200 ps, the amplitude of the center‐of‐mass oscillation increases dramatically under the 1.65 THz resonant field. In contrast, the non‐resonant 10.0 THz field elicits no significant change in amplitude compared to the no‐field condition. This demonstrates a clear resonant excitation of the ion pair's collective motion and provides direct evidence of a non‐thermal manipulation mechanism. From an energetic standpoint, we calculated the 1D Potential of Mean Force (PMF) along the permeation axis (Figure 4b). To ensure statistical reliability, the PMF profiles were derived from the average of three independent simulations, with error bars indicating the standard deviation. A secondary vertical axis representing energy in units of thermal energy (*k_B_T*) was included to explicitly contextualize the barrier heights relative to thermal fluctuations. The resonant 1.65 THz field induces a profound change in the energy landscape, substantially reducing the potential barrier between the two binding sites to approximately 0.3 eV (∼14.6 *k_B_T*). For comparison, the barrier height under the non‐resonant 10.0 THz field is 0.67 eV (∼33.7 *k_B_T*), and it is 0.63 eV (∼32.3 *k_B_T*) in the absence of an external THz electromagnetic waves (EMW) field. This significant reduction in the energy barrier, coupled with the widened potential wells, indicates that the resonant field facilitates ion movement between sites. Collectively, both analyses confirm that applying a THz field resonant with the ions' intrinsic in‐phase oscillation enhances ion permeation, a process achieved by directly driving the collective motion of the bound ions themselves.

Under the 3 V/nm electric field tuned to 1.65 THz, we performed a statistical analysis based on five independent 60 ns simulation trajectories for each condition. As shown in Figure 4c, the average permeation count increased from 0.40 events in the no‐field control (95% bootstrap CI: 0.00–0.80) to 3.20 events (95% bootstrap CI: 2.00–4.20). Statistical evaluation using an exact test for equality of Poisson rates under equal exposure confirmed that this enhancement is highly significant (*p* = 0.0013). This corresponds to a statistically significant enhancement of the average ionic current, rising from ∼2.13 pA to ∼17.07 pA. To confirm that this enhancement was driven by resonance, control simulations using a non‐resonant 10 THz field of the same amplitude yielded 0.80 events / 60 ns (95% bootstrap CI: 0.40–1.00), showing no statistically significant difference from the baseline (*p* = 0.6875). This robust increase in conductance, consistent with our dynamic and energetic analyses, confirms that a resonant THz field can effectively modulate ion channel activity by amplifying the axial oscillations of bound ions.

It is worth noting that two distinct field strengths were employed in our study to address specific research purposes. The relatively low field strength of 0.3 V/nm was used first for the analysis of system dynamics and potential energy landscapes to capture the resonant coupling excitation mechanism. Conversely, a higher field strength of 3 V/nm was adopted for the subsequent multi‐ion permeation simulations. This stronger field serves as a proof‐of‐concept strategy to amplify the resonance coupling effect, allowing us to observe statistically distinguishable permeation events within the accessible simulation timescales (60 ns) by overcoming Poisson noise. Regarding biological safety, while 3 V/nm exceeds the safety thresholds for continuous‐wave THz radiation due to thermal risks, high‐intensity pulsed THz radiation offers a potential avenue for practical physiological experiments. The high peak field of a pulse can induce the resonant effect to enhance ion permeation, while the low duty cycle prevents thermal damage to biological tissues. Furthermore, recent experimental studies have demonstrated that the non‐thermal THz modulation of voltage gated calcium channels is achievable in realistic biological settings, suggesting that the cumulative effect of resonant coupling over physiological timescales may enable observable permeation enhancement even at lower, safer field intensities [38, 58].

#### 2.2.2 Manipulation of Coherence Degree

In addition to enhancing permeation, the external THz field serves as a tool to modulate the coherence of the ion pair's motion. We systematically investigated this phenomenon, along with the influence of ambient temperature, by tracking the Magnitude Squared Coherence (MSC) values specifically at the two identified collective mode frequencies: 1.65 THz (in‐phase) and 2.84 THz (out‐of‐phase). To ensure statistical robustness, data points were derived from the average of three independent simulation trajectories for each condition, with error bars representing the standard deviation.

**FIGURE 5:**
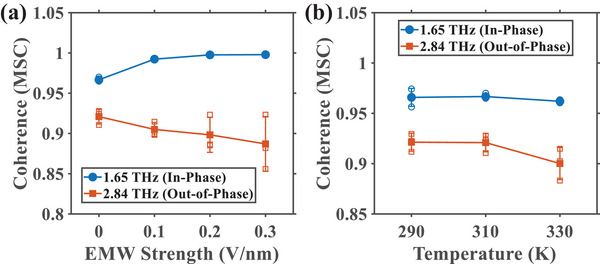
Statistical analysis of the influence of external THz‐EMW strength and temperature on ion‐ion coherence. (a) Magnitude Squared Coherence (MSC) values for the in‐phase mode (1.65 THz, blue circles) and out‐of‐phase mode (2.84 THz, vermilion squares) as a function of resonant field strength (0 to 0.3 V/nm). The resonant field selectively enhances the coherence of the in‐phase mode while suppressing the out‐of‐phase mode. (b) MSC values for both modes at varying temperatures (290, 310, and 330 K) in the absence of an external field. Thermal fluctuations at higher temperatures degrade the coherence of both collective modes. Data are presented as the mean ± standard deviation derived from three independent simulations for each circumstance.

First, we examined the effect of varying the resonant field (1.65 THz) strength from 0 to 0.3 V/nm. As shown in Figure 5a, the coherence of the primary in‐phase mode (blue circles) systematically increases with the applied field strength, exhibiting a saturation trend at higher intensities. Conversely, the coherence of the secondary out‐of‐phase mode (vermilion squares) exhibits a downward trend. This indicates that the external field, being frequency‐matched to the in‐phase motion, selectively promotes its phase synchronization while simultaneously disrupting the coherence of the out‐of‐phase mode. Next, we assessed the role of ambient temperature, which governs the intensity of thermal fluctuations that can disrupt the ions' correlated motion (Figure 5b). As the temperature increases from 290 to 330 K, the coherence of both modes exhibits a decreasing trend, with the out‐of‐phase mode being more susceptible to thermal noise. This confirms that a less disruptive thermal environment (lower temperature) allows the bound ions to maintain a more stable and coherent collective oscillation, whereas higher temperatures introduce random collisions that degrade this correlation.

### 2.3 Quantum Mechanism Analysis of Confined Ions Motion

**FIGURE 6:**
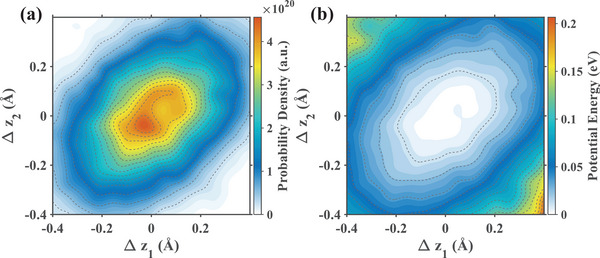
Joint probability density distribution and 2D potential of mean force (PMF) derived from equilibrium dynamics. (a) Position distribution of the two ions' axial displacements (Δz_1_ and Δz_2_) from their respective mean positions over the entire simulation trajectory. Data are accumulated from the simulation trajectory at thermal equilibrium. The probability density is color‐coded, with vermilion indicating regions of highest probability. (b) The corresponding 2D potential of mean force (PMF), representing the effective potential energy profile of the two‐ion system. The PMF is derived from the probability density in (a) via the Boltzmann relation. The color scale represents the potential energy in electron volts (eV), where the white blue central region indicates the potential energy minimum (stable binding state).

Our classical dynamics simulations have provided a detailed picture of the axial motion of the bound Ca^2+^ ions. To gain deeper physical insight, and recognizing that ionic motion in such highly confined environments can exhibit quantum mechanical properties [53, 59, 60], we next transitioned to a quantum mechanical analysis. Crucially, to capture the intrinsic quantum states determined by the channel structure, this analysis was built upon dynamics trajectories obtained at thermal equilibrium (without external THz fields or transmembrane voltage). First, we calculated the joint probability density distribution of the ion pair as a function of their axial displacements (Δz_1_ and Δz_2_) from their respective mean positions. The resulting distribution is shown in Figure 6a. The map is distinctly elliptical and elongated along the main diagonal (where Δz_1_≈Δz_2_). This diagonal represents in‐phase motion, where both ions are displaced from their equilibrium points in the same direction simultaneously. The elongation, therefore, provides direct visual evidence that the ion pair has a strong preference for moving in‐phase. From this equilibrium probability distribution, we derived the two‐dimensional potential of mean force (PMF) via the Boltzmann relation, as depicted in Figure 6b. The PMF represents the effective energy landscape of the coupled two‐ion system. It features an elliptical potential well whose minimum (white region) corresponds to the highest probability region (vermilion region) in Figure 6a. Crucially, the major axis of this ellipse is also aligned with the main diagonal. This confirms that the in‐phase configuration is not only the most probable but also the most energetically favorable state for the collective motion of the ion pair.

**FIGURE 7:**
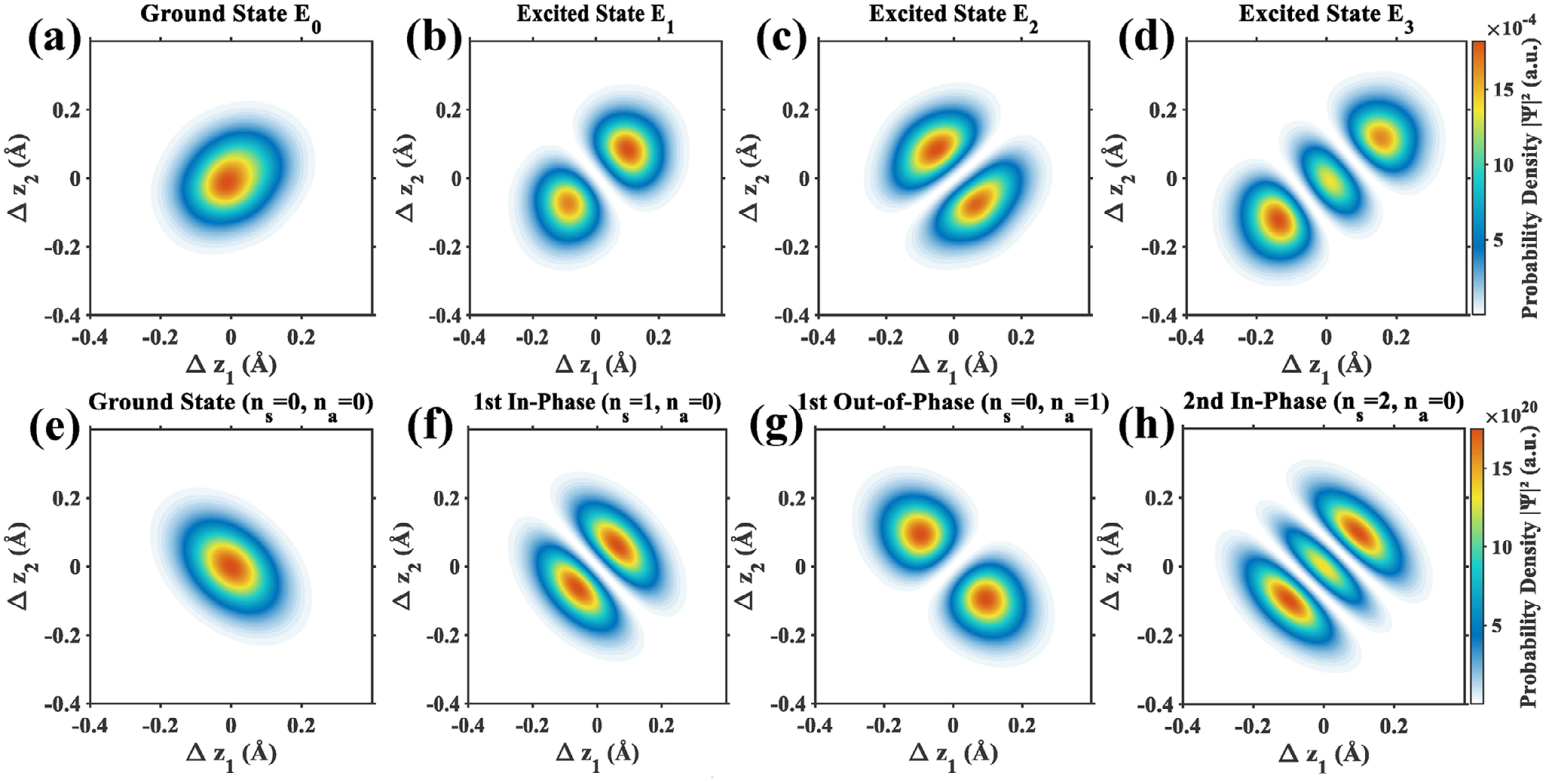
Comparison of quantum states derived from the equilibrium PMF with an ideal coupled quantum harmonic oscillator model. (a‐d) Probability density distributions for the ground state (E_0_) and the first three excited states (E_1_‐E_3_) of the two‐ion system. These wavefunctions are the numerical solutions to the Schrödinger equation using the 2D‐PMF as the potential term. (e‐h) Probability density distributions for the corresponding states of an ideal 2D coupled quantum harmonic oscillator (CQHO) model. These states are labeled by their symmetric (n_s_) and anti‐symmetric (n_a_) quantum numbers. These ideal quantum states can serve as fingerprints to illustrate the modes of different excited states in the two‐ion system. The color scale represents the probability density, where vermilion regions indicate high probability and blue regions indicate low probability. The probability density along the main diagonal represents the in‐phase mode, while the probability density along the anti‐diagonal represents the out‐of‐phase mode.

Using this equilibrium 2D PMF as the potential energy term, we constructed the Hamiltonian for the coupled two‐ion system and solved the corresponding time‐independent Schrödinger equation. This analysis yielded the quantized energy eigenstates and their transition frequencies. Notably, the calculated transition frequencies from the ground state to the first and second excited states are 1.61 THz and 2.60 THz, respectively. These values are in good agreement with the two primary frequencies of 1.65 THz and 2.84 THz identified in our classical dynamics simulations, providing a theoretical quantum mechanical validation of our earlier findings. The probability density distributions for the ground state and the first three excited states are visualized in Figure 7a–d. The first excited state (E1, Figure 7b) exhibits two probability lobes aligned along the main diagonal, corresponding to the in‐phase oscillation mode. In contrast, the second excited state (E2, Figure 7c) displays two lobes along the anti‐diagonal, representing the out‐of‐phase oscillation mode. To deeply interpret these states, we compared them with the eigenstates of an ideal 2D coupled quantum harmonic oscillator (CQHO), which serve as a “fingerprint” for identifying the oscillation modes (Figure 7e‐h). The Hamiltonian for this ideal model shares the exact same kinetic energy term as our system (derived from the dynamics simulations) but utilizes an elastic harmonic potential in place of the complex PMF. The remarkable one‐to‐one correspondence between the PMF‐derived states and the ideal CQHO states confirms our assignments. For example, the pattern of the third excited state (E3, Figure 7d) perfectly matches that of the ideal second in‐phase state (Figure 7h). This comprehensive analysis demonstrates that the collective axial motion of the confined ion pair exhibits discrete quantum eigenstates in our model, and its key characteristics observed in classical simulations are directly mirrored by the underlying quantum eigenstates.

## 3 Conclusions

In this study, we investigated the collective dynamics of confined Ca^2+^ ions within the SF of Ca_v_Ab channel and demonstrated their manipulability by resonant THz waves. We identified intrinsic collective oscillation modes of the confined ion pair: an in‐phase mode at 1.65 THz and an out‐of‐phase mode at 2.84 THz. Simulation results demonstrate that an external electric field tuned to 1.65 THz selectively excites the in‐phase collective motion, remarkably lowering the energy barrier between binding sites and enhancing calcium ions permeation influx at a statistically significant level, while simultaneously allowing the coherence degree to be tuned by the resonant field strength and ambient temperature. By constructing a quantum Hamiltonian on the effective potential landscape derived from dynamics simulations, we obtained quantized eigenstates whose transition frequencies and wavefunction distributions showed good agreement with the classical results, confirming the underlying quantum nature of the observed phenomena in our model.

While our findings highlight the significant potential of resonant coupling, we must acknowledge the limitations of our current model. With the static modeling framework along with a relatively high transmembrane voltage, our results are currently positioned as a proof‐of‐concept demonstration. However, the resonant frequencies and collective vibration modes are determined mainly by the EEEE locus within the selectivity filter. Since this structural motif is highly conserved in human Ca_v_ channels, the proposed resonant mechanism may be theoretically relevant across mammalian physiology. Based on this robustness, the proposed mechanism could be validated in the future through frequency‐resolved electrophysiology (e.g., patch‐clamp Ca^2+^ current recordings of Ca_v_Ab or mammalian Ca_v_ channels under dynamic voltage clamp), combined with high‐intensity pulsed THz radiation to mitigate thermal effects and dedicated waveguides to avoid the strong attenuation of THz radiation in water‐rich biological tissues.

In conclusion, our findings present two significant advances. First, we establish a novel ion‐aimed approach for manipulating Ca_v_ channel function by directly targeting the collective motion of the permeating ions, providing a theoretical foundation for exploring potential physics‐based bio‐electromagnetic strategies. Second, we provide a new physical framework for understanding the high‐flux nature of ion transport, complementing the classical knock‐on model with a mechanism based on the quantized collective motion of the confined ions. By explicitly connecting the ions motion to their quantum eigenstates, our work builds a crucial theoretical bridge between the classical and quantum mechanical descriptions of ion channel dynamics. Our findings not only advance the understanding of ion transport mechanisms from classical and quantum perspectives but also establish a novel conceptual framework to modulate ions permeation in voltage gated calcium channels.

## 4 Methods

### 4.1 Langevin Dynamics

The simulations were based on a model of the Ca_v_Ab channel, with location parameters for the EEEE locus obtained from its PDB entry (ID: 4MVQ) [13]. The charges for the glutamate residues (−1.3 × 10^−19^ C) and dipoles (0.6 × 10^−19^ C) were adopted from established models [9, 55]. The axial (z‐axis) dynamics of the confined Ca^2+^ ions were governed by the Langevin equation:

(1) $$m\;\frac{d^{2}z}{dt^{2}}=F_{cons}\;-\gamma \frac{dz}{dt}+\xi \left(t\right)$$

where m is the mass of a Ca^2+^ ion, *F_cons_* is the sum of the electrostatic and short‐range wall force (detailed derivations are provided in Note S1) [61]. γ is the axial damping rate, which is calibrated against all‐atom molecular dynamics simulations (Note S3). ξ(*t*) is the random force, and all terms strictly correspond to the axial (*z*) coordinate, as this is the coordinate of ion permeation, while ignoring the radial motions (x, y‐axis). When the external THz electric field is applied to the system, Equation (1) can be extended as:

(2) $$m\;\frac{d^{2}z}{dt^{2}}=F_{cons}\;-\gamma \frac{dz}{dt}+\xi \left(t\right)+qE_{z}\left(t\right)$$

where *E_z_* (*t*) is the axial electric field strength. The above equations are numerically integrated and discretely updated using the BAOAB algorithm (detailed in Note S2).

### 4.2 Statistical Analysis

Permeation events were treated as rare count data collected over equal exposure across conditions (N = 5 independent trajectories, 60 ns per trajectory for each condition). Group differences were assessed using an exact test for equality of Poisson rates under equal exposure, implemented as a two‐sided conditional binomial test on the total event counts. The between‐condition effect sizes were quantified as a rate ratio (RR) with exact 95% confidence intervals (Clopper–Pearson inversion under the same conditional framework):

- Resonant vs Control: RR = 8, 95% CI: [1.88, 71.72];
- Resonant vs Non‐resonant: RR = 4, 95% CI: [1.29, 16.44];
- Non‐resonant vs Control: RR = 2, 95% CI: [0.28, 22.11];

We note that the RR confidence intervals are wide because the total event count in the control condition is small (rare‐event regime), and the exact CI is conservative; nonetheless, the RR lower bounds for the resonant comparisons remain >1, consistent with a statistically supported enhancement.

### 4.3 Coherence Analysis

Magnitude squared coherence (MSC) was adopted to evaluate the phase stability of two ions’ velocities at different frequencies, which could be calculated as:

(3) $$C_{12}\;\left(f\right)=\frac{{\left|P_{12}\left(f\right)\right|}^{2}}{P_{11}\left(f\right)P_{22}\left(f\right)}\;$$

where *P*_11_(*f*) and *P*_22_(*f*) are self‐power spectral densities of two ions respectively, and *P*_12_(*f*) is cross‐power spectral density, whose phase describes the average phase difference between two velocities at one frequency.

We extracted the axial acceleration of two ions, characterizing the spectrum of coherent radiation as the square of the sum of axial accelerations, while the spectrum of incoherent radiation expressed as the sum of the square of each individual axial acceleration, delivering as:

(4) $$S_{coh}\propto {\left|\vec{a_{1}}+\vec{a_{2}}\right|}^{2}$$

(5) $$S_{incoh}\propto {\left|\vec{a_{1}}\right|}^{2}+{\left|\vec{a_{2}}\right|}^{2}$$

(6) $$\alpha =\frac{S_{coh}}{S_{incoh}}\;$$

The radiative coherence enhancement factor was expressed as the ratio of two power. When the factor equals to 1, it is denoting that there is no relationship between two ions, as the dashed line in Figure 3c; when the factor values 2, it means that two accelerations are totally same, both in magnitude and direction; when the factor values 0, two accelerations are same in magnitude, but opposite in direction.

### 4.4 Calculation of Quantum Mechanics

The 2D PMF was calculated from the logarithm of the ion probability density distribution based on the Boltzmann relation: [54, 62, 63, 64]

(7) $$U\left(z_{1},z_{2}\right)=-k_{B}T\ln P\left(z_{1},z_{2}\right)$$

where *P*(*z*_1_,*z*_2_) stands for the joint probability density derived from the axial trajectories from control simulations at thermal equilibrium, without any applied transmembrane voltage or external THz field. In these equilibrium runs, the two Ca^2^^+^ ions are confined at binding sites and allowed to relax until their joint axial coordinates (*z*_1_, *z*_2_) sample the stationary distribution. Consequently, *U*(*z*_1_,*z*_2_) represents the intrinsic energy landscape created by the channel's structural features (the EEEE locus) and the ion‐ion interactions. In view of the 2D PMF, the Hamiltonian was constructed as:

(8) $$\hat{H}=-\frac{\hbar^{2}}{2m}\frac{d^{2}}{dz^{2}}+U\left(z_{1},z_{2}\right)$$

As a comparison, the Hamiltonian of ideal 2D coupled quantum harmonic oscillator was:

(9) $$\;\hat{H}=-\frac{\hbar^{2}}{2m}\frac{d^{2}}{dz^{2}}+\frac{1}{2}m_{Ca}{\left(2\pi f\right)}^{2}\Delta z^{2}$$

*f* was set to 1.65 THz and 2.84 THz to represent in‐phase and out‐of‐phase mode.

## Funding

This research was supported by National Natural Science Foundation of China (Nos. T2241002 to S.W., 62527804 To Y.G., 62427806 to Y.L. and 62501129 to Y.S.), Sichuan Province Innovative Talent Funding Project for Postdoctoral Fellows and Center for HPC, University of Electronic Science and Technology of China.

## Conflicts of Interest

The authors declare no conflicts of interest.

## Supporting information




**Supporting File 1**: advs74432‐sup‐0001‐SuppMat.docx.

**Supporting File 2**: advs74432‐sup‐0002‐Figures_Data.zip.

## Data Availability

The data that support the findings of this study are available from the corresponding author upon reasonable request.
