# Supplementary material for: Enhancement of Calcium Ion Permeation via Resonant Coupling of Ion and Terahertz Waves in Voltage Gated Calcium Channels
Source: Adv Sci (Weinh). 2026 Feb 19;13(24):e20475. doi: 10.1002/advs.202520475 (PMC13116260; doi:10.1002/advs.202520475)
Supplement: Supplementary file 1 — Supporting File 1: advs74432‐sup‐0001‐SuppMat.docx. [file ADVS-13-e20475-s001.docx]

Supporting Information

**Enhancement of Calcium Ion Permeation via Resonant Coupling of Ion and Terahertz Waves in Voltage Gated Calcium Channels**

*Zihao Zhang, Yuankun Sun*, Xinqiao Zhou, Sunchao Huang, Shengpeng Yang, Shaomeng Wang* and Yubin Gong**

**Note S1: Components of the conservative force**

The total conservative force, $F_{cons}$, is a sum of forces from the channel and from inter-ionic interactions. The force from the channel includes two components. First, an electrostatic force derived from a pre-computed potential field. This potential arises from the fixed charges of the channel's residues and dipoles, as well as the induced charges generated by their polarization on the protein-water channel wall and calcium ions themselves.

| $U\left( r \right)=\frac{1}{4\pi\varepsilon_{0}\varepsilon_{r}}\left( \sum_{k=1}^{N} \frac{q_{k}}{\left\vert r-r_{k} \right\vert}+\sum_{j=1}^{M} \frac{\sigma_{j}\Delta S_{j}}{\left\vert r-r_{j} \right\vert} \right)$ | (S1) |
| --- | --- |

where, $q_{k}$ and $r_{k}$ are the charge and position of $k^{th}$ fixed charge, and $\sigma_{j}$, $r_{j}$, $\Delta S_{j}$ are the induced charge density, center position, and area on the $j^{th}$ boundary element. $\varepsilon_{r}$ is the effective relative dielectric constant of the water medium inside the channel. In this work, we adopted a value of 8 (rather than the bulk water value of 80). On the one hand, this accounts for the dielectric saturation effect and the restricted rotational mobility of water molecules within the narrow, highly charged selectivity filter. On the other hand, $\varepsilon_{r}$ in formula (3) was utilized to calculate the electrostatic potential energy experienced by the calcium ions, which should be the local dielectric property of the immediate solvent environment of confined Ca^2+^ ions.^61^ Considering the discrete nature of computers, the central difference method is used to calculate the electric field and electrostatic force on each calcium ion, which is the fundamental of potential wells of binding sites.

| $F_{1}\left( r \right)=-q_{Ca}\nabla U(r)$ | (S2) |
| --- | --- |

Second, a short-range repulsive force, described by a Lennard-Jones-like potential,^9^ prevents ions from penetrating the physical channel wall.

| $F_{2}\left( z \right)=\left\vert F_{0}\frac{{(R_{ion}+R_{wall})}^{10}}{{(R_{c}\left( z \right)-a+R_{wall})}^{10}} \right\vert\cdot(-\vec{r})$ | (S3) |
| --- | --- |

$F_{0}$ is a constant, equaling to 10^-9^ N. $R_{ion}$ and $R_{wall}$ are effective radius of the ions and the atoms that make up the channel walls, equaling to 0.99×10^−10^ m and 1.5×10^−10^ m respectively.

Besides the force exerted by the channel, there are Coulomb force and short-range force between calcium ions themselves. Coulomb force expresses as follows:

| $F_{3}\left( r \right)=\frac{1}{4\pi\varepsilon_{0}\varepsilon_{r}}\frac{q_{Ca}^{2}}{\left\vert r_{i}-r_{j} \right\vert^{3}}(\vec{r_{i}}-\vec{r_{j}})$ | (S4) |
| --- | --- |

The short-range force between ions is expressed as a complex empirical formula:

| $F_{4}=-C_{0}\left[ -9\frac{R_{C1}^{9}}{\left\vert r_{i}-r_{j} \right\vert^{10}}+\frac{e^{\frac{R_{C2}-\left\vert r_{i}-r_{j} \right\vert}{C_{3}}}}{C_{3}}\cdot\cos\left( \frac{2\pi\left( R_{C2}-\left\vert r_{i}-r_{j} \right\vert\right)}{d_{w}} \right)-\frac{2\pi e^{\frac{R_{C2}-\left\vert r_{i}-r_{j} \right\vert}{C_{3}}}}{d_{w}}\cdot sin(\frac{2\pi\left( R_{C2}-\left\vert r_{i}-r_{j} \right\vert\right)}{d_{w}}) \right]$ | (S5) |
| --- | --- |

Where, $d_{w}$ equals to $2.76\times{10}^{-10}$ m. $C_{0}$ is $0.8\times k_{B}\times T$, $T$ and $k_{B}$ are 310K and the corresponding Boltzmann constant. $C_{1}$, $C_{2}$, and $C_{3}$ equal to $1.6\times{10}^{-10}$ m, $1.8\times{10}^{-10}$ m and $1\times{10}^{-10}$ m. The empirical parameters above are all from the ref.9 and ref.55. These forces together constitute the conservative force on the confined ions.

**Note S2: Discrete update scheme and time-step dependence of the noise term**

The Langevin equation was numerically integrated using the BAOAB algorithm, which is widely recognized for its numerical stability and thermodynamic consistency to Langevin equations. The exact discrete update scheme implemented in our code is as follows. $\Delta t$ is the time step, and the evolution from step $n$ to $n+1$ consists of the following sub-steps:

(i) B (Velocity Update 1): $v_{n+\frac{1}{2}}=v_{n}+\frac{\Delta t}{2m}F_{cons}(z_{n})$

(ii) A (Position Update 1): $z_{n+\frac{1}{2}}=z_{n}+\frac{\Delta t}{2}v_{n+\frac{1}{2}}$

(iii) O (Ornstein-Uhlenbeck): $\tilde{v}_{n+\frac{1}{2}}=e^{-\gamma\Delta t}v_{n+\frac{1}{2}}+\mathcal{R}_{n}\sqrt{\frac{k_{B}T}{m}(1-e^{-2\gamma\Delta t})}$, where $\mathcal{R}_{n}$ is a random number drawn from a standard normal distribution, $T$ and $k_{B}$ are 310K and the corresponding Boltzmann constant.

(iv) A (Position Update 2): $z_{n+1}=z_{n+\frac{1}{2}}+\frac{\Delta t}{2}\tilde{v}_{n+\frac{1}{2}}$

(v) B (Velocity Update 2): $v_{n+1}=\tilde{v}_{n+\frac{1}{2}}+\frac{\Delta t}{2m}F_{cons}(z_{n+1})$

Considering the O substep, we employ the exact analytical solution of the Ornstein-Uhlenbeck process over the time interval $\Delta t$. The velocity is updated as:

| $v\left( t+\Delta t \right)=v_{t}e^{-\gamma\Delta t}+\sigma\mathcal{R}_{n}$ | (S6) |
| --- | --- |

where $\mathcal{R}_{n}$ is the standard normal random number, and $\sigma$ is the noise amplitude. To satisfy the Fluctuation-Dissipation Theorem (FDT), the integration scheme must preserve the canonical Maxwell-Boltzmann velocity distribution. Specifically, if the velocity variance at time $t$ is at thermal equilibrium $\left\langle{v(t)}^{2} \right\rangle={k_{B}T}/m$, the variance at $t+\Delta t$ must remain ${k_{B}T}/m$. Squaring the equation S1 and taking the ensemble average:

| $\left\langle{v\left( t+\Delta t \right)}^{2} \right\rangle=\left\langle{v(t)}^{2} \right\rangle e^{-2\gamma\Delta t}+\sigma^{2}\left\langle{\mathcal{R}_{n}}^{2} \right\rangle+2\sigma\left\langle v(t)\mathcal{R}_{n} \right\rangle e^{-\gamma\Delta t}$ | (S7) |
| --- | --- |

Since the characteristic of the standard normal random number $\mathcal{R}_{n}$, we substitute the equilibrium variance:

| $\frac{k_{B}T}{m}=\frac{k_{B}T}{m}e^{-2\gamma\Delta t}+\sigma^{2}$ | (S8) |
| --- | --- |

then the exact discretized noise amplitude is obtained:

| $\sigma=\sqrt{\frac{k_{B}T}{m}(1-e^{-2\gamma\Delta t})}$ | (S9) |
| --- | --- |

This method of discretization explicitly includes the time-step dependence ($\Delta t$), ensures the dynamics correctly sample the canonical ensemble at fixed temperature, and satisfies the fluctuation-dissipation theorem. For small time steps ($\gamma\Delta t\ll1$) used in our simulations, $\sigma$ reduces to the standard form:

| $\sigma=\sqrt{2\gamma\frac{k_{B}T}{m}\Delta t}$ | (S10) |
| --- | --- |

which recovers the standard Langevin noise expression.

**Note S3: Damping-rate estimation from all-atom MD and sensitivity analysis**

The standard diffusion-based calculation for $\gamma$ is introduced by:

| $\gamma_{0}=\frac{k_{B}T}{mD}$ | (S11) |
| --- | --- |

which typically predicts overdamped motion for ions, and contradicts the distinct oscillatory behavior observed in atomistic simulations. To bridge this gap, a short all-atom molecular dynamics simulation of confined Ca^2+^ ions in the selectivity filter of the Ca_v_Ab channel (PDB ID: 4MVQ) was carried out using GROMACS at 310 K, and a 100-ps equilibrium trajectory was recorded. Based on the axial velocity of the bound ion, the normalized velocity autocorrelation function (VACF) was computed as:

| $C_{vv}\left( \tau\right)=\frac{\left\langle v_{z}(t)v_{z}(t+\tau) \right\rangle}{\left\langle v_{z}^{2}(t) \right\rangle}, C_{vv}\left( 0 \right)=1$ | (S12) |
| --- | --- |

Then, we obtained the power spectral density (PSD) of the VACF and each resonance peak was fitted with a simple Lorentzian line shape, corresponding to the near-resonance form of the damped-oscillator spectrum:

| $S\left( \omega\right)=\frac{\gamma}{{(\omega^{2}-\omega_{0}^{2})}^{2}+{(\gamma\omega)}^{2}}$ | (S13) |
| --- | --- |

Fig. S1. Power spectral density of the axial Ca^2+^ velocity calculated by GROMACS. Black dots show the velocity power spectrum obtained from the equilibrium trajectory, and the red line is the double-Lorentzian fit. The excellent agreement between data and fit confirms that the dominant spectral features are well described by two underdamped harmonic modes, and the corresponding linewidths (FWHM) are used to estimate the effective damping rates in our Langevin model.

Fig. S2. Sensitivity of the axial acceleration spectra to the damping-rate scaling factor. (a), (c), (e) Power spectra of the axial acceleration of the ion at the 14 Å binding site for scaling factors $\alpha=0.01, 0.02, 0.04$. (b), (d), (f) Corresponding spectra for the ion at the 18 Å binding site. Changing $\alpha$ mainly modifies the peak height and linewidth, while the positions of the dominant resonances remain essentially unchanged, indicating that the identified collective modes are robust against reasonable variations of the effective damping rate.

The Lorentzian fitting yields a damping rate of $\gamma_{1}=1.42\times{10}^{11} s^{-1}$. Therefore, the dimensionless scaling factor was defined as:

| $\alpha=\frac{\gamma_{1}}{\gamma_{0}}$ | (S14) |
| --- | --- |

which serves to restore biological realism to the simplified model, ensuring it reproduces the oscillation nature of the ion actually observed in the atomistic environment.

To assess robustness, we have performed repeating the Langevin dynamics simulations for several values of the scaling factor ($\alpha=0.01, 0.02, 0.04$). The results, summarized in Fig. S2, show that changing within this physically reasonable range mainly affects the quality factor and peak height of the resonant response, while the locations of the dominant resonance frequencies remain essentially unchanged. Thus, the axial resonant frequency (1.65 THz) of the confined ions is an intrinsic property of the channel's potential energy landscape (specifically the curvature of the EEEE binding site wells), robust against variations in the damping rate.

Further, we performed simulations across a frequency range of 1.0 THz to 3.0 THz to characterize the frequency response of the permeation rate. Specifically, we selected seven frequencies (1.00, 1.30, 1.65, 2.00, 2.30, 2.60, and 3.00 THz) and conducted three independent permeation simulations for each frequency under the same protocol used in the main text. The resulting frequency-response curve is summarized in Fig. S3.

Fig. S3. Frequency response of Ca²⁺ permeation events. The average number of permeation events (derived from three independent 60-ns simulations per frequency) is plotted as a function of the external field frequency. Error bars represent the bootstrap 95% confidence interval of the mean. The response curve exhibits a pronounced peak at the intrinsic in-phase mode frequency of 1.65 THz, indicating the resonant coupling effect.

Notably, the permeation enhancement exhibits a pronounced maximum at 1.65 THz, where the average permeation events reach a maximum (~4.33 events), offering the evidence for the resonant coupling mechanism.

**Note S4: Structural stability analysis of the Ca_v_Ab channel**

To access the conformational stability of the Ca_v_Ab channel and justify the use of average potential energy landscape of our Langevin model, we performed a 100-ns all-atom molecular dynamics simulation using GROMACS. The model was built with the pore region of CavAb (PDB entry: 4MVQ) (residues 130-219) embedded in 1-palmitoyl-2-oleoyl-glycero-3-phosphocholine (POPC) bilayer to mimic the cellular membrane environment. The system was solvated with an aqueous solution containing CaCl_2_ with an ionic concentration of 0.15mol/L. The TIP3P model was used for water molecules. After the energy minimization, the simulations were conducted with the method of V-rescale to scale the temperature to the bath of 310K (NVT equilibration). And C-rescale was adopted to maintain the pressure constant at 1 bar (NPT equilibration). Once the system is equilibrated sufficiently, semi-isotropic Parrinello-Rahman barostat algorithms replaced the C-rescale. The detailed simulation parameters and settings can be found in Table. S1.

Table. S1. The force constants for different objects being restrained in different simulation stages.

| Stage | Time | Position restraints | | | |
| --- | --- | --- | --- | --- | --- |
|  |  | Protein | | Lipids | |
|  |  | Backbone  (kJ/mol/nm^2^) | Sidechain  (kJ/mol/nm^2^) | P atom  (kJ/mol/nm^2^) | Dihedral  (kJ/mol/nm^2^) |
| EM | / | 4000 | 2000 | 1000 | 1000 |
| NVT | 125 ps | 4000 | 2000 | 1000 | 1000 |
| NVT | 125 ps | 2000 | 1000 | 400 | 400 |
| NPT | 125 ps | 1000 | 500 | 400 | 200 |
| NPT | 500 ps | 500 | 200 | 200 | 200 |
| NPT | 500 ps | 200 | 50 | 40 | 100 |
| NPT | 500 ps | 50 | 0 | 0 | 0 |
| NPT | 100 ns | 0 | 0 | 0 | 0 |
| MD | 100 ns | 0 | 0 | 0 | 0 |
|  | 100 ns | 200 | 50 | 1000 | 400 |
|  |  | Excluding SF residues | | | |

Fig. S4. Comparative structural stability analysis of the Ca_v_Ab channel. (a, b) Global Backbone Stability: Time evolution of the RMSD for the entire protein backbone under (a) unrestrained and (b) restrained conditions. The unrestrained system shows stable thermal equilibrium (~0.22 nm). (c, d) Intrinsic Stability of the Selectivity Filter (SF): Time evolution of the RMSD specifically for the SF residues (EEEE locus) under (c) unrestrained and (d) restrained conditions. Note that the SF maintains an extremely low RMSD (~0.07 nm) in both cases, confirming that the binding site geometry is structurally stable and robust against global protein motions.

To estimate the conformation of the protein, we calculated the Root Mean Square Deviation (RMSD) of the protein backbone, which is an important quantity to estimate the geometrical deviation, and is widely used to determine the equilibration of the protein. As depicted in Fig. S4(a), the backbone RMSD of the entire Ca_v_Ab channel rapidly reaches a plateau and remains within a narrow range throughout the 100-ns trajectory, indicating no large-scale conformational transitions beyond thermal fluctuations. Crucially, when we extract the RMSD of the selectivity filter residues from this same trajectory, the value drops significantly to ~0.07 nm (Fig. S4(c)). Furthermore, we conducted another 100-ns backbone-restrained simulation (excluding the SF), in which the RMSD of the backbone fluctuates around 0.07 nm (Fig. S4(b)), equaling to the RMSD of SF under the unrestrained circumstance. This consistency indicates that the global thermal fluctuations (0.22 nm) are primarily driven by the flexible loops and termini, while the SF core remains structurally conserved. Also, we extracted the RMSD of the SF residues with backbone-restraint, as shown in Fig. S4(d). The RMSD exhibits a nearly unchanged mean and a narrower fluctuating range, which reveals that the SF geometry is relatively stable. These results support that, while the thermal fluctuations are present, the core structure of the selectivity filter remains relatively stable over the simulation timescale. Consequently, the potential energy landscape derived from the Langevin model, which is based on the stable conformation, serves as a physically justified approximation for capturing the intrinsic resonant properties of the confined ions in the binding sites.
